# Continuous Glucose Measurements for Diet Monitoring in Healthy Adults

**DOI:** 10.1177/19322968251361555

**Published:** 2025-08-12

**Authors:** Linda Ong, Claudine J. Lamoth, André van Beek, Ming Cao, G. J. (Bart) Verkerke, Elisabeth Wilhelm

**Affiliations:** 1 Engineering and Technology Institute Groningen, University of Groningen, Groningen, The Netherlands; 2 Department for Human Movement Science, University Medical Center Groningen, University of Groningen, The Netherlands; 3 Department of Endocrinology, University Medical Center Groningen, University of Groningen, The Netherlands; 4 Department of Rehabilitation Medicine, University Medical Center Groningen, University of Groningen, The Netherlands

**Keywords:** CGM metrics, continuous glucose monitoring, food diary, healthy adults, linear mixed model, population without diabetes

## Abstract

**Background:**

Lifestyle interventions and low glycemic diets have potential in diabetes prevention. However, dietary monitoring relies on self-report, which is prone to under-reporting. This observational study investigated the correlation between continuous glucose monitoring (CGM) metrics and glycemic load (GL) or daily macronutrients consumption.

**Methods:**

Based on one week of CGM data, actigraphy measurements, and food diaries, we investigated correlations between GL per meal, and 19 CGM metrics, selected based on 20 studies identified via a systematic literature review. Furthermore, we generated linear mixed models to predict GL and macronutrients intake using moderately correlated CGM metrics.

**Results:**

Forty-eight healthy participants (27 women, average age of 28.2 years, average body mass index (BMI) of 23.4 kg/m^2^) were included. We found significant positive moderate correlations (*P* < .0004) between GL and area under the curve (ρ = 0.40, two-hour window), relative amplitude (ρ = 0.40, three hours and ρ = 0.42, four hours), standard deviation (SD) (ρ = 0.41, four hours), and variance (ρ = 0.43, four hours). Significant positive moderate correlations (*P* < .0004) were observed between carbohydrate and SD (ρ = 0.45), variance (ρ = 0.44), and mean amplitude of glycemic excursions (MAGE) (ρ = 0.40) over 24 hours. We obtained one valid mixed linear model for predicting GL from CGM metrics obtained two hours after food intake. A second model predicted energy intake using moderately correlated CGM metrics, body composition, sleep duration, and physical activity.

**Conclusion:**

We demonstrated moderate correlations between GL and CGM metrics in healthy populations. These CGM metrics were successfully used to predict GL or energy intake.

## Introduction

Low glycemic load (GL) diets might be beneficial for preventing diabetes type 2.^1^ Tracking of GL could support people to maintain such diets. However, under unconstrained eating conditions GL is usually estimated using food diaries. These diaries are known to underestimate the actual food intake.^2^ Photography-based diaries, that estimate energy and carbohydrate intake, reduce the burden of the user.^3^ However, the user engagement they require still leads to intentional and unintentional under-reporting.^4^

Wearables for continuous glucose monitoring (CGM) could be used to estimate carbohydrate intake for health and self-optimization.^5^ Recent studies demonstrated that these sensors can record postprandial glucose profiles in healthy populations.^6-8^ However, algorithms that connect CGM data with GL or macronutrients are needed before CGM sensors can be used for diet monitoring.

### Related Work

Based on a systematic search on PubMed, Embase, and Web of Science using the search term “continuous glucose monitoring glycemic load.” 251 potentially related studies were identified. After duplicate removal based on title by Excel, 188 papers remained. Title and abstract screening were performed by one author. Original research papers relating CGM measurements to meals with a specific glycemic index (GI) or to GL obtained from food diaries were included. Studies in children, in animals, and studies containing pharmacological interventions were excluded. 20 relevant studies were identified and summarized in Table 1.

**Table 1.** Previous Studies Exploring the Relationship Between Dynamic CGM Metrics and Food Intake.

Studies with standardized meals					
Source/Population	Participants	Duration	Extracted CGM metrics	Window	Goal
Kizirian et al^9^ pregnant women	17	2 times 24 h	MAGE, AUC, SD, TIR, TAR, TBR	24 h	compare diets
Tramunt et al^10^ gastric bypass patients	56	3 days	maximum, peak	postprandial	linear model CGM GL + carbs
Aston et al^11^ overweight women	12	3 d	AUC, mean, SD	24 h	compare diets
Waldenmaier et al^12^ healthy adults	30	2 d	AUC	1 - 5 h	compare diets
Pearce et al^13^ patients with diabetes	23	4 times 3 d	MOOD AUC	5 h 20 h	food intake timing
Maitland et al^14^ obese pregnant women	16	6 d	mean	3 h	compare drinks
Camps et al^15^ healthy adults	15	2 times 24 h	iAUC, tAUC, maximum, TIR, MAGE	24 h 2 h	compare diets
Buscemi et al^16^ obese healthy population	24	3 months	mean, CV, SD	48 h	compare diets
Zavitsanou et al^17^ diabetes type 1	12	6 times 5 h	tAUC, peak, TTP	5 h	compare specific meals
Sato et al^18^ young adults	10	2 times 24 h	TTP, AUC AUC	5 h sleep	compare timing
Grout et al^19^ diabetes type 2	25	2 times 8.5 h	raw values	24 h	cognitive function vs glycemic response
Fechner et al^20^ obese / overweight	8	2 times 12 h	tAUC, CONGA4	2 h	compare diets
Henry et al^21^ healthy adults	20	2 times 24 h	MAGE, AUC, iAUC	24 h	specific dietary product
Lagerpusch et al^22^ healthy men	32	6 weeks	iAUC, AUC	24 h	compare diets
Zahedani et al^23^ patients with diabetes + healthy	665	10 days	TIR	240 h	app validation

**Table:** 

Studies with unconstrained meals					
Population	Participants	Duration	Extracted CGM metrics	Window	Goal
Fabricatore et al^24^ obese diabetes patients	26	3 days	MAGE, mean, SD, TIR, TAR	24 h	correlation CGM GL
Van Baak^25^ obese healthy population	25 (field) 9 (lab)	72 h	Mean, SD SD CONGA 1	48 h Day/night 24 h	compare diets
Jardine et al^26^ pregnant women with diabetes	29	3 days	iAUC mean	24 h 3 h	correlation CGM GL physical activity
Tsujino et al^27^ healthy adults	27	4 days	mean, TTP	various windows	establish population CGM profile
Philippou et al^28^ healthy adults	18	12 weeks	AUC	24 h overnight	compare diets

Most studies investigated effects of different diets^9,11,12,15,16,20,22,25,28^ or specific food items^14,17,20^ on dynamic characteristics of CGM curves. Other studies explored the effect of timing of food intake on glucose dynamics.^13,18^ Three studies tried to establish statistical models relating GL to CGM measurements.^10,24,26^ However, these studies investigated specific patient population, which limits their generalizability.

Most studies provide standardized food or supervise meals. Studies with unsupervised food intake using food diaries or 24-hour recall as reference are less frequent. One study with a mixed approach reported significant differences in glucose variability measures such as continuous overlapping net glycemic action over one-hour periods (CONGA1) depending on dietary GI in the lab that could not be verified in the field.^25^

A broad variety of mathematical CGM metrics can be found in the scientific literature. Only one paper used the raw CGM values to differentiate between a high and a low glycemic response group.^19^ Papers that use standard parameters of descriptive statistics such as mean, standard deviation (SD), and the coefficient of variance (CV) often do not report significant results.^11,16^ Significant differences between diets are often reported for AUC,^17,21^ obtained using the trapezoidal function.^17^ Some papers distinguish between the baseline corrected incremental area under the curve (iAUC) and the total area under the curve.^15^

In addition to general statistic parameters, some metrics that have been specifically designed for CGM analysis. Glucose variability in studies with significant findings is often described by the mean amplitude of glycemic excursions (MAGE).^21^ MAGE is defined as the arithmetic mean of glucose increases (nadir to peak) or decreases (peak to nadir) when both exceed one standard deviation of 24-hour CGM. While MAGE originally was defined on 48-hour window,^29^ most studies apply it on 24-hour measurements. The mean of the daily differences (MODD) describes the interday variance. It is defined as the mean of the absolute difference between two blood glucose values selected with respect to meal time. However, in diet comparison studies this parameter was not significant.^13^ For diabetes research, CONGAn, defined as the standard deviation of the difference between the current glucose readings and the glucose readings n hours prior,^30^ is also applied. A commonly used CGM metric in diabetes management is time in range (TIR), which refers to the percentage of time a person’s glucose levels remain within a defined target range. The current international consensus recommends using 70 to 180 mg/dL (3.9-10.0 mmol/L) as the standard euglycemic range for most nonpregnant adults with diabetes. However, some studies have adopted alternative thresholds based on specific research objectives or patient populations. In addition to TIR, time above range (TAR) has been proposed as a complementary CGM metric to assess hyperglycemia.^24^ Several studies also report a metric known as time to peak (TP), which typically refers to the time interval between a glucose-raising event (such as a meal) and the subsequent peak glucose concentration. However, definitions of “peak” vary across studies: some consider the maximum glucose value within a given period^17,31^ while others define all values exceeding a certain threshold—for example, 140 mg/dL—as peaks.^10^ In individuals without diabetes, glucose levels usually remain within the euglycemic range, and such excursions above 140 mg/dL are uncommon.

Studies reporting correlations between CGM and GL in patient populations used different CGM metrics. In gastric bypass patients, a dependency between postprandial peaks in CGM and carbohydrate content of food has been observed.^10^ In patients with diabetes a significant partial correlation GL and SD (*r* = 0.41) and MAGE (*r* = 0.38) on 24-hour observation periods after controlling for total energy intake was reported.^24^ In pregnant women, correlations between GL and iAUC or three-hour postprandial glucose were not significant.^26^ Due to the use of various variables and diverse outcomes, it remains unclear which CGM metrics are best suited for predicting GL from CGM measurements, especially in people without diabetes.

### Objective

This study investigated the correlation between GL and CGM metrics and examined whether CGM metrics can predict GL or macronutrient content of unconstrained food intake in healthy participants.

## Methods

### Participants

Adults without diabetes and eating disorders aged between 25 and 34 years were eligible to participate in the study. Exclusion criteria were pregnancy and presence of electric body implants. These exclusion criteria were necessary due to the use of a bioimpedance scale. Additional exclusion criteria were taking any prescribed medication besides contraceptives or being unable to understand instructions in either English or Dutch. According to an a priori sample size calculation based on CGM values reported for healthy individuals,^7^ the recruitment target was 48 participants. Details of the sample size calculation are included in the Supplementary Material.

The study was carried out in accordance with the declaration of Helsinki. The Medical Ethical Committee stichting Biomedisch Onderzoek Assen waived the necessity of obtaining medical ethical approval under the Dutch law governing medical research with human subjects (23.108/CW). The institutional ethics committee (CETO) of the Faculty of Arts, University of Groningen, reviewed the experiment protocol and had no objection to the proposal (ID96048183). Written informed consent was obtained from all participants prior to enrollment.

### Study Design

Each participant participated seven consecutive days in this observational study.

After obtaining informed consent, personal information (age and biological sex) was collected via a questionnaire. Afterwards, the waist circumference was recorded. Body mass index (BMI), body fat, and muscle mass were measured using the bioimpedance scale Tanita RD 545 (Tanita, Tokyo, Japan). Participants registered at the Mijn Eetmeter application (https://www.voedingscentrum.nl). Then participants received a pre-configured smart wristband GENEActiv (Activinsights, Cambridgeshire, UK) used to measure their physical activity and sleep quantity continuously. The wristband was worn on the nondominant wrist, unless participants requested otherwise. The glucose sensor (FreestyleLibre 2; Abbott, California, USA) was attached to the back their nondominant upper arm.

Participants recorded food and drinks they consumed grouped by breakfast, lunch, dinner, and snack in the Mijn Eetmeter app. They scanned the glucose sensor before consuming meals and noted the main meal type. Participants were also asked to scan the sensor once before sleep to prevent data loss.

### GL Calculation

The GI of the different types of food consumed by the participants was obtained from the 2021 version of the international table of GI.^32^ The GI of N individual food items (GI_food_) in combination with the amount of carbohydrates (gCarbs_food_) was used to calculate the total GL per meal (MealGL) using equation (1):^19^

(1) $$\begin{array}{c} MealGL=\sum_{i=1}^{N}GI_{food\;i}\times g\frac{Carbs_{food\;i}}{100} \end{array}$$

### CGM Metrics

Based on literature, we calculated the AUC, mean, SD, MAGE, and CV. Furthermore, we calculated the time between food intake and the highest local maximum (*peak*) in CGM. To avoid confusion with threshold-based TPs, we name this CGM metric rise-time $(t_{rise}).$ Variance, skewness, kurtosis, and slope were added as recommended by a recent publication on machine learning–based glucose-level forecasting.^33^ The rebound of the glucose curve after the peak might also be influenced by the carbohydrate intake. Therefore, the fall-time (*t_fall_*) defined as the time between the global maximum and the following local minimum, was included and AUC and slope were calculated for the rise (*AUC_rise_* and *slope_rise_*) and the fall period (*AUC_fall_* and *slope_fall_*). The relative amplitude (*Amp*), defined as the difference between the highest CGM value and the CGM at the time of food intake, was also recorded. Furthermore, we registered the smallest CGM value in the observation period (*Amp_min_*). From classical signal analysis we added the ratio between rise and fall slope (*slope_ratio_*) and the steepest slope (*slope_steepest_*).

### Statistical Analysis

Shapiro-Wilk tests and Q-Q plots were used to determine whether the data were normally distributed. The homoscedasticity of the model residual was tested by using the Levene test. The results of these tests are presented in the Supplementary Material. Correlations were analyzed using Pearson correlation, or Spearman correlation if data were not normally distributed. In total, we computed 119 correlations. Therefore, according to the Bonferroni correction alpha is set to 0.0004 for the correlation analysis. All analyses were performed with Python 3.9 using the libraries numpy, statistics, statsmodel, and scipy.stats.

### Outcome Measures

The main outcome measures of the study were the correlation between GL and 19 CGM metrics obtained on two-hour, three-hour, and four-hour windows, and the correlation between macronutrients and six CGM metrics (AUC, mean, SD, MAGE, CV, and Var) over 24-hour periods starting at midnight. Macronutrients included energy in kCal and carbohydrates, fiber, and sugar in grams.

The secondary outcomes of the study were seven linear mixed models (LMMs). Three models predict GL from CGM variables and four models predict macronutrients intake over 24 hours. We included CGM metrics that showed at least moderate correlation (ρ ≥ 0.40) as independent variables. Each model was once tested with and once without personal characteristics such as demographics and body composition. Physical activities and sleep duration were included in some models as they might influence food intake.^34^ Participants were set as random variables in our LMM models. For valid models we reported the equation, log-likelihood, SD error, and residual of the models, and the important variables. Furthermore, the mean and standard deviation (std) of mean absolute error (MAE) from leave one out cross validation (LOOCV) was presented to provide some insight of prediction error when models are tested blindly in different participants and the fluctuation of their error, respectively.

## Result

### Participants

Fifty participants participated in the study. Two participants dropped-out on the second day due to discomfort caused by CGM. Three participants needed to replace the glucose sensors on the first day, due to sensor detachment during high-intensity physical activities or to sensor malfunction during attachment, resulting in mild bleeding. In total, 48 healthy participants (21 men, 27 women), completed the full measurement period. Table 2 shows the demographics of the study population.

**Table 2.** Demographic Table for Sleep Quantity, Body Composition, and Physical Activities.

Category	Characteristics	Units	Mean (std)		
			All (n = 48)	Female (n = 27)	Male (n = 21)
Body Composition	Waist Circumference	cm	81.4 (10.7)	77.4 (11.7)	86.5 (6.7)
	Muscle mass	kg	51.8 (9.4)	45.6 (6.1)	59.7 (6.6)
	Body fat	%	23.6 (6.5)	26.3 (6.9)	20.1 (4.0)
Sleep	Duration	hours	6.1 (2.3)	6.5 (2.2)	5.7 (2.3)
Physical Activity	Sedentary		9.7 (3.4)	9.7 (3.4)	10.0 (3.2)
	Light		2.5 (2.1)	2.5 (2.1)	2.4 (2.1)
	Moderate		2.4 (1.6)	2.3 (1.4)	2.6 (1.8)
	Vigorous		0.09 (0.2)	0.06 (0.1)	0.11 (0.25)

For all participants, the mean of CGM values was below 6.2 mmol/L. The median, min, and max of the within- individual CV was 16.1%, 11.6%, and 25.6%, respectively. Two of these participants experienced one hyperglycemic event (CGM > 11.1 mmol/L) and one participant experienced three hyperglycemic events. One participant had a CV of 20.8% in combination with an average CGM of more than 5.8 mmol/L. Shah et al^35^ reported that in a cohort of healthy, adults without diabetes, the mean CGM glucose was consistently below 6.2 mmol/L, and the within-individual coefficient of variation (CV) ranged from 11.6% to 25.6%, with a median of 16.1%. While a few participants had transient hyperglycemic readings or CVs above 20%, their mean CGM values remained below 5.8 mmol/L. These findings indicate that such CGM profiles are still compatible with nondiabetic physiology. However, CGM metrics alone (mean glucose and CV) are not sufficient to diagnose or exclude diabetes, and clinical diagnosis requires established criteria such as fasting glucose, oral glucose tolerance test, or HbA1c.

### Exploratory Analysis

In total, 5040 entries were recorded in MijnEetmeter. Among these were 1651 unique types of food components. Only for 732 out of these food components GI could be extracted from the scientific tables. If the GI value of one component in a meal was unknown, this particular meal was excluded from the GL analysis. Eventually, 210 main meals with GL were extracted. Meals without a scientifically validated GL value were still included in the macronutrients analysis.

A total of 595 main meals, 292 snacks, and 101 drinks were recorded together with CGM data. To synchronize CGM data with the food diaries, food diary entries were matched with the annotations made by the participants while scanning the glucose sensors.

After synchronization, 120 main meals with GL information from 26 participants could be matched with CGM data. One main meal was excluded as the z-score of GL exceeded 3. This outlier was related to scanning a family pack of food without declaring the portion size. Depending on the window size, five to nine meals had to be excluded as there was insufficient data to extract the CGM metrics.

Daily macronutrients such as energy, carbohydrate, fiber and sugar, were completely reported for 352 days from 48 participants. Two datapoints were excluded as the z-score of energy from one of the recorded meals exceeded 6. The food diary showed an unrealistic big portion size suggesting that the participant mistakenly entered the wrong unit or the quantity of food consumed. For example, once the unit “table spoon” was mistakenly selected instead of “grams.” Furthermore, some data sets were excluded due to missing data in the activity recording leaving 329 data points for this analysis.

### Correlation

Table 3 shows the correlation between GL and CGM metrics from 26 participants. Significant positive moderate correlations were observed between GL and AUC (ρ = 0.40, *P* < .0004, two hours), Amp (ρ = 0.40, *P* < .0004, three hours, and ρ = 0.42, *P* < .0004, four hours), SD (ρ = 0.41, *P* < .0004, four hours), and Var (ρ = 0.43, *P* < .0004, four hours).

**Table 3.** Correlation Between Glycemic Load and Continuous Glucose Monitoring (CGM) Over Different Time Windows From 26 Participants.

CGM metric	Glycemic load				
	2h	3h	4h	5h	6h
*AUC*	.40*	.32	.27	.16	.06
*AUC_rise_*	.07	.06	.00	–.06	–.18
*AUC_fall_*	.11	.14	.14	.16	.20
*Amp*	.36	.40*	.42*	.35	.20
*Ampmin*	.28	.26	.14	.21	.06
*trise*	.02	.01	–.04	–.06	–.19
*tfall*	–.09	.00	.03	.03	.13
*slope_rise_*	.34	.37	.29	.24	.24
*slope_fall_*	–.32	–.26	–.20	–.21	–.10
*sloperatio*	.22	.12	.04	.04	.03
*slopeavg*	.16	.17	.15	.09	.04
*slopesteepest*	.03	.13	.22	.21	.06
*Mean*	.38	.38	.38	.35	.30
*SD*	.30	.36	.41*	.37	.28
*MAGE*	.36	.32	.36	.33	.25
*CV*	.23	.30	.35	.31	.22
*VAR*	.34	.37	.43*	.38	.29
*Skew*	.04	.05	–.13	–.22	–.20
*Kurtosis*	.03	–.10	–.31	–.35	–.29

* *P* value = 1.21 x ^10-5^ (AUC_2h_), 1.31 x ^10-5^ (Amp_3h_), 3.58 x ^10-6^ (SD_4h_), 3.19 x ^10-6^ (Amp_4h_), 1.27 x ^10-6^ (Var_4h_).

The correlation between macronutrients and CGM measurements from 48 participants is depicted in Table 4. Significant positive moderate correlations were observed between carbohydrate and glucose variability of SD (ρ = 0.45, *P* < .0004), MAGE (ρ = 0.40, *P* < .0004) and Var (ρ = 0.44, *P* <.0004).

**Table 4.** Correlation Between Macronutrients and CGM Over 24-Hour Periods From 48 Participants.

CGM metric	Glycemic load			
	Energy	Carbohydrate	Fiber	Sugar
AUC	.35	.34	.27	.27
Mean	.29	.29	.26	.13
SD	.34	.45*	.30	.30
MAGE	.30	.40*	.30	.30
CV	.28	.37	.24	.27
VAR	.34	.44*	.30	.30

* *P* value = 1.78 x ^10-18^ (SD_carbs_), 4.52 x ^10-15^ (MAGE_carbs_), 2.77 x ^10-18^ (Var_carbs_).

### Linear Mixed Models

#### GL prediction models

We generated models for two-hour, three-hour, and four-hour observation periods with or without personal characteristics. Independent variables included AUC, Amp, and Var. Standard deviation was not included in the model because it was strongly correlated with Var (ρ = 1.0, *P <* .05) and it had lower correlation to GL than Var.

**Figure 1.**
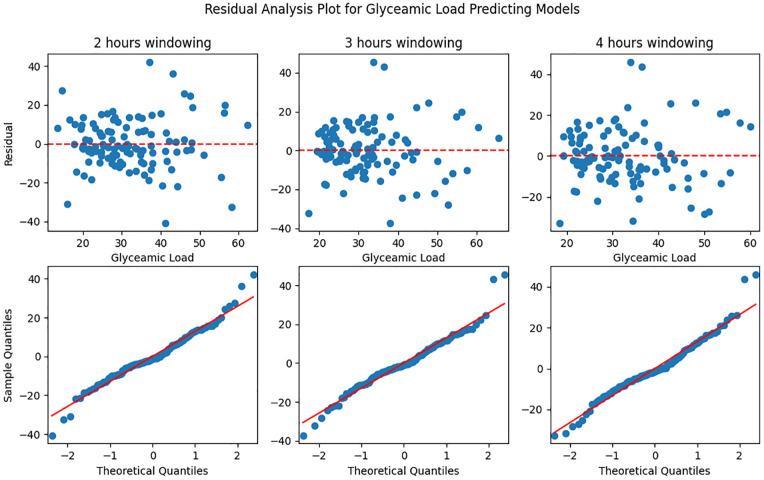
Residual analysis plot for glycemic load predictive models without personal characteristics in two, three, and four hours windowing. Top plot: predictive glycemic value vs residual; bottom plot: Q-Q plot.

We found one valid model for predicting the GL in a window of two hours without personal characteristics. The model was homoscedastic (*P* < .05) and the residual of the model was normally distributed (*P* = .07). The Q-Q plots and plots of the residuals over the predictive values for GL predictive models without personal characteristics can be found in Figure 1. In the model with a window of two hours, AUC and Var were important glucose characteristics (*P* < .05). This LMM model is described in equation (2) where *e_id_* is the random error:

(2) $$\begin{array}{c} \begin{array}{l} MealGL_{2h}=-23.0+0.001\times AUC-3.56\;\;\times \\ \;\;\;\;\;\;\;\;\;\;\;\;\;\;\;\;\;\;\;\;\;\;\;\;\;\;\;\;Amp+16.7\times Var+e_{id} \end{array} \end{array}$$

$$e_{id}\sim N(0,1)$$

The mean and standard deviation (std) of MAE from LOOCV for predicting GL of meal intake in two-hour windowing are 14.2 and 9.1, respectively.

The other GL prediction models were invalid due to non-normally distributed residuals.

#### Macronutrient prediction models

We generated models for predicting energy, carbohydrate, sugar, or fiber intake with or without personal characteristics using the CGM metrics MAGE and SD. Var was not included as an independent variable due to a high correlation with SD (ρ = 1.0, *P* < .05).

Only the model predicting energy intake including personal characteristics had normally distributed residuals (*P* = .07) and was homoscedastic. Equation (3) describes this model:

(3) $$\begin{array}{c} \begin{array}{l} Energy\left(kCal\right)=162.3+7.7\times BiologicalGender+\\ \;\;\;\;\;\;\;\;\;\;\;\;\;\;\;\;\;\;\;\;\;\;\;\;\;\;\;\;\;\;\;\;\;\;\;\;410\times ModeratePA+97.2\times \end{array}\\ SedentaryPA-12.7\times Age-3.5\times WaistCirumference\\ +\;30.1\times MuscleMass-13.9\times Bodyfat+\\ 0.008\times Sleep+848\times SD-21.1\times MAGE+e_{id} \end{array}$$

$$e_{id}\sim N(0,1)$$

The mean and std of MAE from LOOCV for predicting energy intake in 24-hour windowing are 473.4 and 185.4 kCal, respectively.

**Figure 2.**
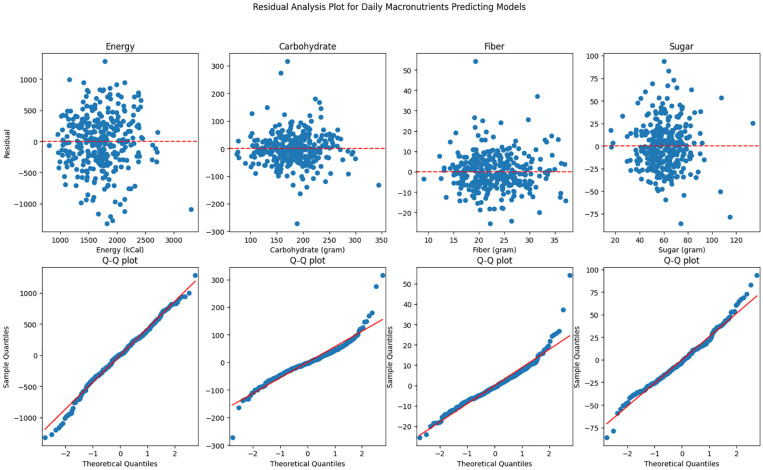
Residual analysis plot for daily macronutrients predictive models with personal characteristics. Top plot: predictive macronutrient value vs residual; bottom plot: Q-Q plot.

The Q-Q plots and plots of the residuals over the predictive values for daily macronutrients predictive models with personal characteristics can be found in Figure 2.

The summary of parameters from two valid predictor models is depicted in Table 5. More detailed information on all models is presented in the Supplementary Material.

**Table 5.** Two Valid Linear Mixed Models for Predicting Glycemic Load in Two Hours Windowing and for Predicting Energy Intake.

Dependent variable	Importance				
	Log-likelihood	Coefficient	(*P* < .05)	*P* value	Std error
GL (2 h)	−469.6	.001	AUC	.00031	13.7
		16.7	Var	.002	
Energy	−2477	848	SD	.001	835
		30.1	Muscle Mass	.003	
		.008	Sleep	.006	

## Discussion

We presented a moderate correlation between CGM metrics (AUC, Amp, SD, and Var) and GL in people without diabetes in an observational study with unrestricted food intake. This is in line with previous studies in patient populations.^10,24,26^ In addition, we were able to build an LMM that predicts GL based on CGM metrics. Notably, the window size used to obtain this model was only two hours which is smaller than the window size reported in patient studies.^26^

Similar to a study that involved obese patients with type 2 diabetes,^24^ we observed significant correlations between carbohydrate content and AUC and mean in the healthy population. In contrast to studies in patients, we found that CGM metrics expressing glucose variability such as SD and MAGE (ρ = 0.40-0.45, *P* < .0004) were also moderately correlated with total carbohydrate intake. This difference might be attributed to a lower glucose variability in healthy populations before eating compared with patients with diabetes.

Furthermore, we found that SD, muscle mass, and sleep were important variables to predict the amount of energy intake. This correlates with literature, reporting a relationship between sleep duration and energy intake.^36^

This study is limited by the small number of meals originating from only 26 participants for which GL could be calculated according to the scientifically validated GI tables. Many commonly consumed ingredients in Dutch diets are not included in those tables. Therefore, the model for predicting the GL is underpowered. This problem can only partially be solved by increasing the number of participants as the limited availability of scientifically validated GI values would still introduce bias in the model. Therefore, we reported additional models and correlations for macronutrient intake. However, that means the number of models increases the risk of type 1 errors and therefore the significance reported for model variables need to be taken with caution.

The low reliability of food diaries, which are the current gold standard, also affected our ground truth recording. Over-reporting of food intake was identified using z-scores and the respective meals were excluded from the analysis. Unfortunately, a similar measure could not be defined for under-reporting as there is no minimum amount of energy people consume at an individual meal. In addition, for several meals the exact time of food intake has not been recorded by the participants. Therefore, the analysis of the postprandial windows has a smaller sample size than the 24-hour analysis. Another limitation of the study arises from the CGM. Due to the necessity of using the Freestyle Libre app for data recording participants were able to see their glucose values. This could have altered their eating and/ or reporting behavior.

No participant reported a diabetes diagnosis. However, CGM measurements of 11 participants exceeded the CV threshold or contained hyperglycemic events. That could mean that we also included participants with pre-diabetes. As diabetes type 2 is a continuum and does not occur suddenly, models for the general population need to be able to deal with such users. Therefore, these participants were included in the analysis.

## Conclusion

We established an LMM based on AUC and Var obtained from two hours postprandial CGM for predicting GL in a healthy population. A second LMM predicted daily energy intake using SD of CGM, sleep duration, and muscle mass. In addition, we could show that AUC, Amp, SD, and Var obtained from CGM are significantly moderately correlated with GL. Significant moderate correlations were also found between SD, VAR and MAGE and the daily carbohydrate intake. This makes CGM a promising tool for diet monitoring in healthy populations. Further studies investigating the effect of age and exploring machine learning–based analysis are needed before CGM can be used as a tool in lifestyle interventions.

## Supplemental Material

sj-docx-1-dst-10.1177_19322968251361555 – Supplemental material for Continuous Glucose Measurements for Diet Monitoring in Healthy Adults Supplemental material, sj-docx-1-dst-10.1177_19322968251361555 for Continuous Glucose Measurements for Diet Monitoring in Healthy Adults by Linda Ong, Claudine J. Lamoth, André van Beek, Ming Cao, G. J. (Bart) Verkerke and Elisabeth Wilhelm in Journal of Diabetes Science and Technology
