# Supplementary material for: Continuous Glucose Measurements for Diet Monitoring in Healthy Adults
Source: J Diabetes Sci Technol. 2025 Aug 12;20(4):1242–51. doi: 10.1177/19322968251361555 (PMC12343543; doi:10.1177/19322968251361555)
Supplement: Supplementary file 1 [file sj-docx-1-dst-10.1177_19322968251361555.docx]

Supplementary material for the paper:

Continuous glucose measurements for diet monitoring in healthy adults

# Linda Ong^1^, Claudine J. Lamoth^2^, Andre´ van Beek^3^, Ming Cao^1^, G.J.(Bart) Verkerke ^4^, Elisabeth Wilhelm^1^

# Sample size calculation

Eq 1 was used to calculate the sample size necessary for a mixed linear model that predicts Glycemic Load from glucose level and other independent factors namely age, Body Mass Index, waist circumference, physical activity intensity, sleep quantity, body fat, and muscle mass. Based on the values Freckman et al. reported for healthy individuals, the minimal clinical significance difference of a glucose level (∆MCD) was set to 10 mg/ dl with a standard deviation (σ) of 17.5 mg/ dl [1]. The significant interval (α) and the power (β), were set to 95% and 80%, respectively. t defined as 1 measurement (within 7 days). The resulting required sample size was 48 participants.

$$\begin{aligned} N=\frac{2\left( \frac{Z_{a}}{2}+ Z_{\beta} \right)^{2}\left( 1+\left[ t-1 \right]\rho\right)}{{t\left( \frac{\Delta_{MCD}}{\sigma} \right)}^{2}}\#\left( 1 \right) \end{aligned}$$

*α* = significant level

*β* = power

*t* = repeated measure

*ρ* = within-subject correlation

∆*_MCD_* = minimal clinical different

*σ* = standard deviation of glucose level in everyday condition

# Linear Mixed Models

a. ***Glycemic Load prediction based on CGM features***

Var was an important feature in the three and four-hour model (*P <* .05). However, residuals of LMM in these two models were not normally distributed (*P <* .05). The Q-Q plots and plots of the residuals over the predictive values for these models can be found in figure1. Parameters of the mixed linear models are summarized in Table 1.

**Figure 1.** Residual Analysis Plot for Glycemic Load predictive Models without personal characteristics in two, three and four hours windowing. Top plot: Predictive Glycemic Value vs Residual; Bottom plot: Q-Q plot

**Table 1.** Linear Mixed Model for predicting Glycemic Load with two to four hours windowing. Independent variables: AUC, Amp, Var; Random variable: 26 Participants

| Window | Log- | Importance | Std | Residual |
| --- | --- | --- | --- | --- |
| Size (h) | Likelihood | (*P <* .05) p-value | Error | (*P >* .05) |
| 2 | -469.6 | AUC .00031 | 13.7 | yes |
| 3 | -474.4 | Var .002  Var *<* .00001 | 12.1 | no |
| 4 | -484.0 | Var *<* .00001 | 10.7 | no |

b. ***Glycemic Load prediction based on CGM and personal characteristics***

To extend the previous models we added personal characteristics such as demographic, body composition, sleep duration, and physical activity levels independent variables obtained from CGM.

There was no statistically significant importance of personal characteristics in predicting GL (*P >*.05) even though AUC and Var still showed significant importance in the model (*P <* .05). Moreover, the residual of the model from two to four hours windowing was not normally distributed (*P <*.05).

c. ***Macro nutrient prediction based on CGM features***

SD showed an importance in predicting Energy, Carbohydrate, and Sugar intake (*P <* .05). However, the residual of the predicting models were not normally distributed (*P <* .05).

d. ***Macro nutrient prediction based on CGM and personal characteristics***

Muscle mass and SD were important variables in predicting amount of carbohydrates (*P <* .05). Muscle mass was an important predictor for the amount of fibers consumed (*P <* .05) while moderate physical activities (PA) and SD were important variables in predicting Sugar (*P <* .05). The Q-Q plots and plots of the residuals over the predictive values for these models can be found in figure 2. Table 2 summarizes the parameters of the mixed linear models for macronutrients that included personal characteristics as independent variables.

**Figure 2.** Residual Analysis Plot for daily Macronutrients predictive Models with personal characteristics. Top plot: Predictive Macronutrient value vs Residual; Bottom plot: Q-Q plot

**Table 2.** Linear Mixed Model for predicting Macronutrients with both CGM features and personal characteristics Independent variables: SD, MAGE; Random variable: 48 Participants

| Macro | Log- | Importance | Std | Residual |
| --- | --- | --- | --- | --- |
| Nutrient | Likelihood | (*P <* .05) p-value | Error | (*P >* .05) |
| Energy | -2477 | SD .001 | 835 | yes |
|  |  | Muscle Mass .003 |  |  |
|  |  | Sleep .006 |  |  |
| Carbs | -1831 | SD .0004 | 112.7 | no |
|  |  | Muscle Mass .028 |  |  |
| Fiber | -1247 | Muscle Mass .014 | 18.9 | no |
| Sugar | -1579 | SD .021 | 49.7 | no |
|  |  | Moderate PA .005 |  |  |
|  |  |  |  |  |

[1]	Freckmann G, Schauer S, Beltzer A, Waldenmaier D, Buck S, Baumstark A, et al. Continuous glucose profiles in healthy people with fixed meal times and under everyday life conditions. J Diabetes Sci Technol 2024;18:407–13.
